# Bringing rehabilitation home with an e-health platform to treat stroke patients: study protocol of a randomized clinical trial (RGS@home)

**DOI:** 10.1186/s13063-022-06444-0

**Published:** 2022-06-20

**Authors:** Anna Mura, Martina Maier, Belén Rubio Ballester, Javier De la Torre Costa, Judit López-Luque, Axelle Gelineau, Stephane Mandigout, Per Hamid Ghatan, Raffaele Fiorillo, Fabrizio Antenucci, Ton Coolen, Iñigo Chivite, Antonio Callen, Hugo Landais, Olga Irina Gómez, Cristina Melero, Santiago Brandi, Marc Domenech, Jean-Christophe Daviet, Riccardo Zucca, Paul F. M. J. Verschure

**Affiliations:** 1 Laboratory of Synthetic, Perceptive, Emotive and Cognitive Systems (SPECS), Institute for Bioengineering of Catalonia (IBEC), Barcelona, Spain; 2 Institut de Recerca Sant Joan de Déu, Barcelona, Spain; 3 Parc Sanitari Sant Joan de Déu, Barcelona, Spain; 4 HAVAE Laboratory EA 6310, University of Limoges, Limoges, France; 5 Uppsala University Hospital (UUH), Uppsala, Sweden; 6 Saddle Point Science Ltd., London, UK; 7 Fondation de l’Avenir pour la recherche médicale, Paris, France; 8 Eodyne Systems S.L., Barcelona, Spain; 9 Medtronic Ibérica S.A., C/María de Portugal 11, Madrid, Spain; 10 Department of Physical Medicine and Rehabilitation, University Hospital Center of Limoges, Limoges, France; 11 Hospital del Mar Medical Research Institute Foundation, IMIM, Barcelona, Spain; 12 Institució Catalana de Recerca I Estudis Avançats (ICREA), Barcelona, Spain

**Keywords:** Randomized clinical trial, Stroke, Virtual reality, Motor recovery, Upper extremities, Wearables, Home treatment, E-health, Deep tech

## Abstract

**Background:**

There is a pressing need for scalable healthcare solutions and a shift in the rehabilitation paradigm from hospitals to homes to tackle the increase in stroke incidence while reducing the practical and economic burden for patients, hospitals, and society. Digital health technologies can contribute to addressing this challenge; however, little is known about their effectiveness in at-home settings. In response, we have designed the RGS@home study to investigate the effectiveness, acceptance, and cost of a deep tech solution called the Rehabilitation Gaming System (RGS). RGS is a cloud-based system for delivering AI-enhanced rehabilitation using virtual reality, motion capture, and wearables that can be used in the hospital and at home. The core principles of the brain theory-based RGS intervention are to deliver rehabilitation exercises in the form of embodied, goal-oriented, and task-specific action.

**Methods:**

The RGS@home study is a randomized longitudinal clinical trial designed to assess whether the combination of the RGS intervention with standard care is superior to standard care alone for the functional recovery of stroke patients at the hospital and at home. The study is conducted in collaboration with hospitals in Spain, Sweden, and France and includes inpatients and outpatients at subacute and chronic stages post-stroke. The intervention duration is 3 months with assessment at baseline and after 3, 6, and 12 months. The impact of RGS is evaluated in terms of quality of life measurements, usability, and acceptance using standardized clinical scales, together with health economic analysis. So far, one-third of the patients expected to participate in the study have been recruited (*N* = 90, mean age 60, days after stroke ≥ 30 days). The trial will end in July 2023.

**Discussion:**

We predict an improvement in the patients’ recovery, high acceptance, and reduced costs due to a soft landing from the clinic to home rehabilitation. In addition, the data provided will allow us to assess whether the prescription of therapy at home can counteract deterioration and improve quality of life while also identifying new standards for online and remote assessment, diagnostics, and intervention across European hospitals.

**Trial registration:**

ClinicalTrials.gov NCT04620707. Registered on November 3, 2020

## Background

Stroke is the second leading cause of death worldwide and the primary contributor to the burden of neurological disease [1, 2]. According to the World Stroke Organization [3], there are over 13.5 million stroke cases every year, of which roughly 40% require rehabilitation, and the total world population of chronic stroke survivors is estimated at 80 million. These growth trends constitute a true crisis for healthcare systems which calls for new solutions to reduce costs, increase sustainability, and obtain better patient outcomes [4]. In addition, the COVID-19 crisis has caused a tremendous strain on stroke care and denied stroke patients necessary rehabilitation [5]. This demonstrates how health crises can deeply disrupt healthcare and the need to create more resilient systems. Digital health services have the potential to directly contribute to answering these challenges. Yet, this does require their alignment with the patient journey and its varying requirements in the clinic and at-home phases of rehabilitation, including the formulation, validation, and adoption of remote interventions and digital health strategies. Telerehabilitation has emerged as a promising solution for stroke rehabilitation, and several studies have shown promising results but also fundamental methodological limitations [6]. Most of these studies are underpowered, do not test or monitor patients at the clinic as inpatients and in their homes, or have follow-up measurements beyond the intervention period. In addition, most studies usually do not evaluate the cost-effectiveness of the intervention [7]. The RGS@home trial aims to address these shortcomings, focusing on the patients’ improvement of independence and quality of life by reducing the limitations of activities of daily living (ADL).

**Fig. 1:**
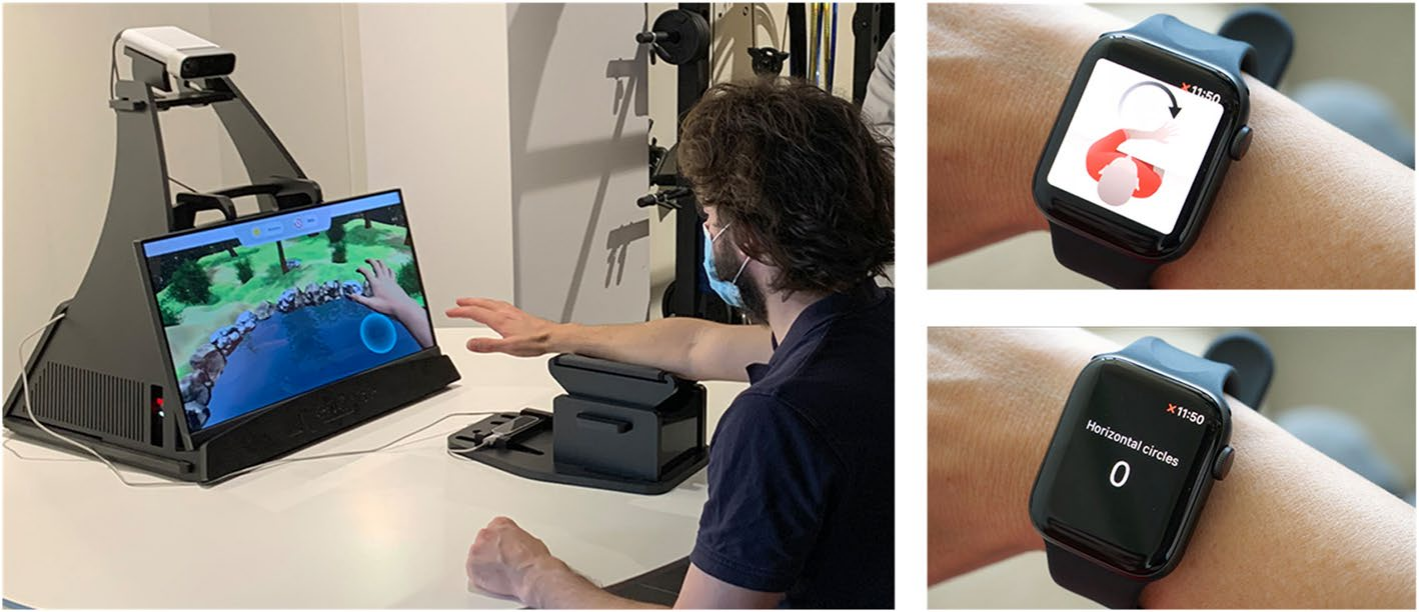
The Rehabilitation Gaming System (RGS) and its use during the patient’s journey to recovery. [Left] RGS consists of a touch screen computer, a motion capture system (Microsoft Kinect) to track the patient’s upper body movements while performing exercises in VR-based scenarios, a Leap Motion camera to track the movement of the hands, and the smartwatch RGSwear (either Fossil, TicWatch Pro, or Apple iWatch, which may be paired or not with a smartphone) to track the movement of the paretic arm. [Right] The RGSwear reminds the patient to perform circular movements every other day and displays the progress of the paretic arm

We have designed and implemented the validation of an e-health rehabilitation intervention for stroke patients and assessed its clinical impact, usability, and acceptance aligned with the patients’ journey to recovery. The technology included in this study capitalizes on RGS (Fig. 1), a cloud-based platform ​for delivering AI-enhanced rehabilitation protocols using virtual reality (VR), motion capture, wearables, and mobile apps that can be used in the hospital and at home. The core principles of the brain theory-based RGS intervention are to construct rehabilitation exercises in the form of embodied goal-oriented and task-specific actions [8]. The RGS platform uses AI techniques to individualize the exercises, VR for their delivery, and cloud and edge computing for data processing and storage. Thus far, interventions using RGS have been shown to be effective in enhancing functional recovery in acute, subacute, and chronic stroke patients [9] in several domains, i.e., motor [10–12], cognitive [13], language, and affect [14]. In addition, chronic stroke patients that trained with RGS improved their performance of ADL compared to a control group, and these changes correlated with cortical reorganization [15, 16].

### Study rationale and hypotheses

**Fig. 2:**
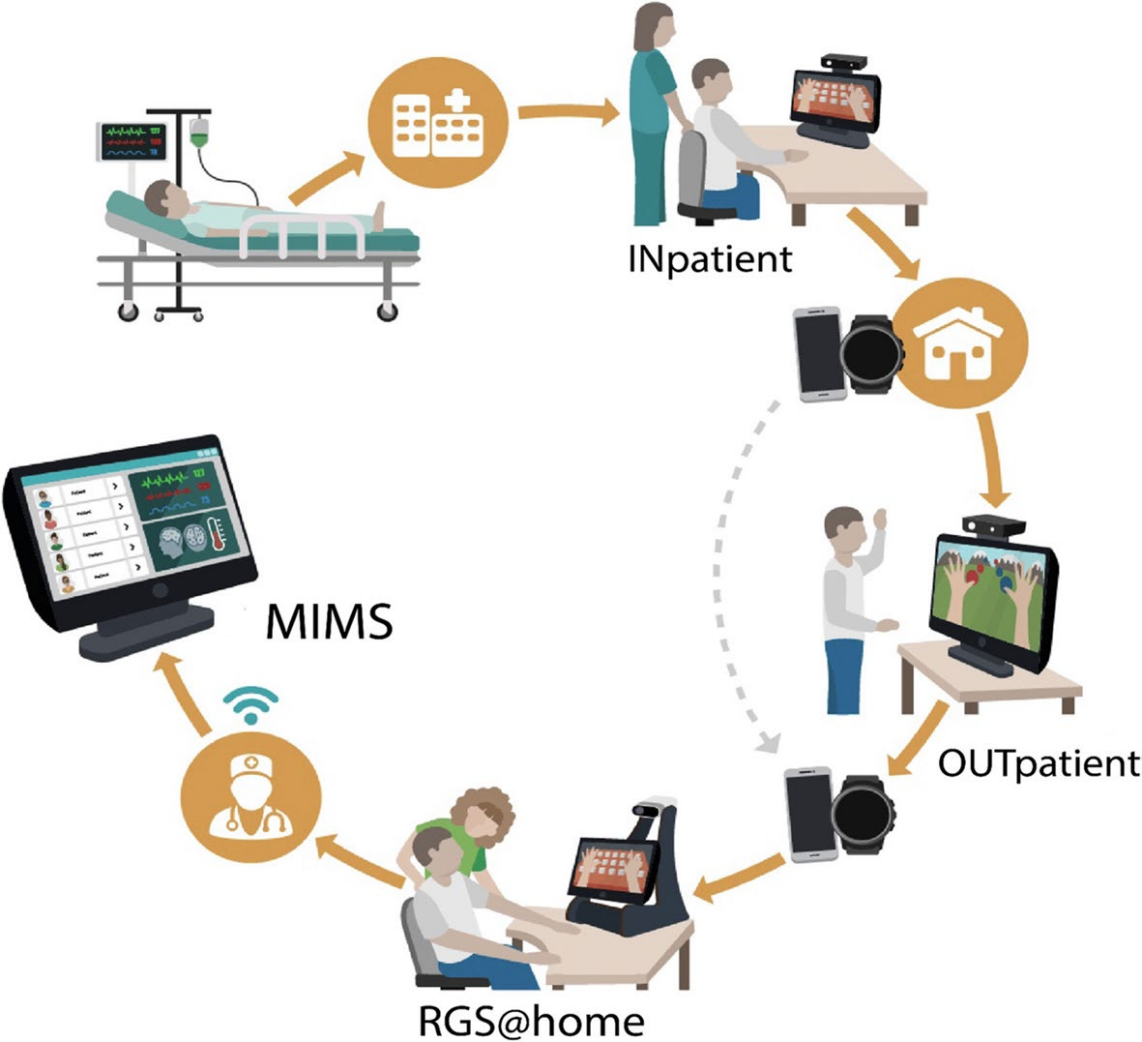
The RGS platform and its use during the patient’s journey to recovery. The RGS grants a continuum of care and rehabilitation, monitoring, and analytics that supports clinicians in their decision-making (Medical Information Management System (MIMS)) and stroke patients (inpatients and outpatients) and with a “soft-landing” from the hospital to home

This study proposes that deterioration after stroke (poor quality of life and function) can be counteracted by enabling the patients to continue their rehabilitation regime at home and monitoring their activities to stay engaged and train daily. We assess the RGS solution’s effectiveness, acceptance, and cost-effectiveness along with post-stroke monitoring and care continuum (Fig. 2). To achieve this goal, we use the RGS platform at the hospital, during the inpatient phase, and at the patients’ homes during the outpatient phase. The RGS platform includes a wearable device, the RGSwear, to track and promote the use of the paretic limb (Figs. 1 and 2).

RGS@home is a multicentric study, and the trial is conducted in three different hospitals across Europe, namely, Spain, Sweden, and France. Thus, this study will also provide insights into whether the proposed system can be used independently of country-specific practice, standards of stroke care, and reimbursement systems. We hypothesize that the RGS setup combined with conventional therapy will be a more effective rehabilitation program than conventional therapy alone. More specifically, we expect the combined approach to show high adherence to treatment and higher effects on function and well-being, while costs will be reduced. A specific effect we seek to reveal is the positive feedback between rehabilitation, ADLs, and recovery, which we see as a process that can overcome deterioration which can be achieved with e-health solutions. We hypothesize that spontaneous use of the paretic limb, as measured with the RGSwear wearable, is predictive of recovery.

## Trial status

The RGS@home protocol was approved by the local Ethical Committee of the three participating hospitals, in Spain, Sweden, and France. Recruitment began in November 2020 and is to be completed in July 2022. The study will end in July 2023. The study was registered on November 3, 2020, at ClinicalTrials.gov (NCT04620707).

## Method

### Study design

The RGS@home study is a randomized controlled trial (RCT) with stroke patients recruited from three hospitals in Spain, Sweden, and France. This RCT compares the impact of the intervention with the RGS platform plus standard care versus standard care alone. The standard care was provided to the patient as recommended in each hospital, on primary and secondary outcomes (see the “Outcome measures” section).

### Participants and recruitment

This RCT includes stroke patients (age 20–85 years old) at the subacute and chronic stages of the disease that are recruited voluntarily among patients admitted to the stroke rehabilitation units of Parc Sanitari Sant Joan de Déu (PSSJD, Barcelona, Spain), Uppsala University Hospital (UUH, Uppsala, Sweden), and the Centre Hospitalier Universitaire de Limoges (LIM, Limoges, France). The study experienced delays in the recruitment process due to the situation enforced in the hospitals by the COVID-19 health crisis.

The RGS@home trial protocol adheres to the international guidelines (Declaration of Helsinki, Edinburgh, 2000; Council of Europe Convention for the Protection of Human Rights and Dignity of Human Being concerning the Application of Biology and Medicine, Oviedo, 1997; Universal Declaration on Bioethics and Human Rights adopted by UNESCO’s General Conference on 19 October 2005).

### Inclusion criteria

Clinicians in each country perform a pre-selection to identify potential patients for the study. Those patients giving consent and signing the informed consent form are screened for inclusion according to the inclusion and exclusion criteria defined in Table 1.

**Table 1:** Inclusion and exclusion criteria

**Inclusion criteria**	
Patients presenting a first-ever ischemic or intracerebral hemorrhagic stroke	
A CT scan and/or MRI to exclude other pathologies	
Lesion localization by clinical symptoms/signs	
Moderate to mild proximal upper limb motor impairment (MRC ≥ 2) and/or moderate to severe non-fluent aphasia (Barcelona test or equivalent)	
Age 20–85 years old	
Able to sit on a chair or a wheelchair interacting with RGS during a full session and be capable and willing to participate in RGS therapy	
**Exclusion criteria**	
Presence of a condition or abnormality that, in the opinion of the investigator, would compromise the safety of the patient or the quality of the data	
Severe cognitive capabilities preventing the execution of the experiment (MoCA < 19), but the final decision is under the clinician’s criterion	
Arteriovenous malformation or lesions not related to a stroke	
Severe associated impairment such as proximal but not distal spasticity, communication disabilities (sensory, Wernicke aphasia, or apraxia), major pain or other neuromuscular impairments, or orthopedic devices that would interfere with the correct execution of the experiment (Modified Ashworth Scale < 3)	
Unable to use RGS independently according to the clinician’s observations and lacking support from a caregiver to use RGS	
Refusal to sign the informed consent form	
Pre-stroke history of upper limb motor disability	

### Allocation and blinding

After being assessed for eligibility, patients are randomly allocated to either the intervention or control group (allocation ratio 2:1). The larger sample size in the experimental group allows more variables to be tested and gives more power to detect relevant effects in the experimental intervention. Randomization is realized via an adaptive stratified sampling (i.e., minimization) through the computerized encrypted cloud-based database (electronic case report form (eCRF)), which takes into account three conditions to balance the two groups: (1) time since stroke (≥ 30 days), (2) age (≥ 20 years old), and (3) severity (≥ 2 MRC). The allocation is concealed while patients and clinicians (except those involved in the baseline assessment) are not blind to the group allocation. A sham group was excluded from the design because an earlier study has shown a superior rehabilitation effect of training with RGS over sham computer game training using the Wii gaming console [10]. Since this is a first-of-a-kind study evaluating the outcomes (see Tables 2 and 3.) of an AI platform for rehabilitation, the sample size has been determined by feasibility (3 years) and estimated by considering the monthly number of eligible inpatients and outpatients of the participating hospitals. We plan to include a total of 90 patients via the clinical partners in the consortium. Each hospital will be recruiting 30 patients, 20 in the intervention group and 10 in the control group.

**Table 2:** Study procedure and assessment

Study procedure	Screening and baseline assessment		Control group, standard care	Intervention group, 0–3 months daily sessions of 24 min or more	Post-treatment assessment		
	Inpatients	Outpatients			3 months	6 months	12 months
Informed consent	x	x					
Medical history	Patients’ demographics MRI/CT scan when available						
Assessment: domains and scales							
Disability and impairment	Barthel Index (BI)				x	x	x
	Stroke Impact Scale (SIS)				x		x
	Fugl-Meyer Assessment of the upper limb (UE-FM)				x	x	x
	Chedoke Arm and Hand Activity Inventory (CAHAI)				x		x
	Hamilton Depression Rating Scale (HDRS)				x		x
	Visual analog score (VAS) for pain				x		x^a^
	Modified Ashworth Scale (AS) for spasticity				x^a^		x
	Fatigue Severity Scale (FSS)				x		x
	Grip force^a^				x^a^		x^a^
	Kinematics from the motion capture camera of the RGS@home system				x	x	x
	RGSwear data (paretic arm)				Continuous		
	Incidents related to use				x		x
	Number and reason of dropouts				x		x
	Acceptability and usability Questionnaire for patients and physicians.				x	x	x
Quality of life	Stroke Specific Quality of Life scale (SSSQOL)				x		x
	Number of falls				x	x	x
	Short-Form-36 (SF-36)				x	x	x

^a^Included in the eCRF but not in the trial registry

**Table 3:** Additional variables collected via eCRF

Assessment		3 months	6 months	12 months
Readmission	Number of outpatients that return to the hospital after discharge	x	x	x
	Reason for readmission	x	x	x
	Days of hospitalization due to readmission	x	x	x
	Costs of readmission	x	x	x
	Presence of CVA risk markers such as waist to hip ratio, smoking cessation, blood pressure, hypertension, and blood markers	x	x	x
Cost of treatment	Total cost of therapy	x		x
	Hours of therapy	x		x
	Total cost of traveling from home to hospital	x		x
	Hours of traveling from home to hospital	x		x
	Number of days of inpatient hospitalization	x		x
Quality of life	Return to work	x	x	x
	Number of formal caregiver hours per week	x	x	x
Acceptability	Number of hours of system use at home	x		x
	Number of hours technical/visits to patients’ home	x	x	x
	Number of equipment replacements	x		x
	Incidents related to use	x		x

### Timeline patients’ assessment

**Fig. 3:**
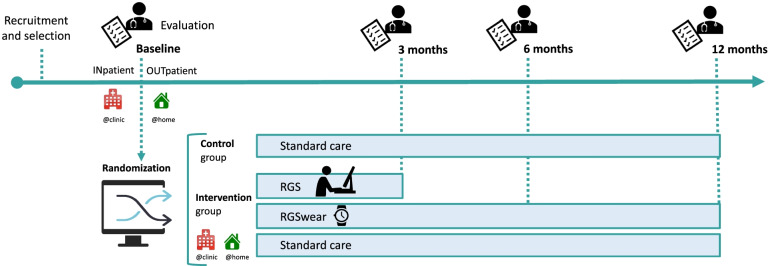
Clinical trial timeline. Participants are recruited as inpatients shortly after a stroke or as outpatients after hospital discharge. After a pre-selection, participants receive baseline evaluations and are randomly assigned to the intervention group or control group. The inpatients start using the RGS desktop setup during their stay at the hospital, while the outpatients use RGS directly in their homes. All participants in the intervention group are provided with a wearable device (RGSwear) for continuous monitoring. The control group receives rehabilitation as usual according to the standard care of the countries included in the study. According to the outcome measures assessments, all participants are evaluated again at 3, 6, and 12 months after baseline evaluation (see Tables 2 and 3)

The RGS@home RCT is a longitudinal study that recruits and assesses stroke patients at different time points after stroke (Fig. 3). All patients are evaluated on primary and secondary outcome measurements by trained clinicians (different from those that performed the baseline assessment) at the respective hospital.

### Study procedure: baseline measures and follow-up assessments

The participants’ characteristics are collected at baseline and include demographic data (age, gender, height, weight, address of rehabilitation center), social situation (marital status, social support at home, occupation status, educational level), and data related to their clinical history (date of stroke, lesion location, paretic side, dominant side). Tables 2 and 3 show the clinical scale used at baseline and at a follow-up to assess motor function, disability, activities of daily living, quality of life, and other measurements, covering the five domains of interest (see outcome measures).

We also collect data from the RGS setup, including the type of protocols selected, the time spent using the protocols, the kinematics obtained with the motion capture imagers, and the users’ performance. The data from the RGSwear wearable is also collected.

### Outcome measures

Outcome measures are collected at four time points during the study (see Fig. 3) and include measurements to assess disability and impairment as well as quality of life (see Table 2). Additional variables are collected via the eCRF (but not included in the trial registry) and include readmission, cost of treatment, quality of life, and acceptance (see Table 3).

#### Primary outcome measures

One main outcome measure is calculated as a compound score summarizing the main outcome of each of the 5 domains: (1) disability and impairment (Barthel Index for independence in activities of daily living), (2) readmission (number of patients who return to the hospital (inpatient) after being discharged to at-home status), (3) cost of treatment (total cost of the therapy), (4) quality of life (the Stroke Specific Quality of Life Scale), and (5) acceptability (amount of use at home).

#### Secondary outcome measures

The secondary outcome of this study is the impact of stroke diagnosis on ADL involving the use of the upper limb. We use the Fugl-Meyer Assessment for Upper Limb (UE-FM), Barthel Index, and the Stroke Impact Scale to quantify these variables.

For the interaction with the RGS system, we measure the daily use, derived estimates of joint synergies, range of motion, reaching speed, precision, etc. (see [17] for details on the analyzed parameters). For the RGSwear, we analyze the daily use based on step count and accelerometer data.

### Intervention and control groups

As explained in the “Allocation and blinding” section, patients are allocated in a randomized fashion either to the intervention group (RGS treatment plus rehabilitation as usual) or to the control group (standard care) (see Fig. 3 and Tables 2 and 3).

The *intervention group* follows a rehabilitation program for motor training using the RGS system at the clinic and home in addition to standard care. The training consists of various RGS-based exercises involving reaching, grasping, and placing virtual objects in a virtual environment. The training period and the number of training sessions are variable. They are set by the treating clinicians based on the patients’ needs and on dosage guidelines agreed by the three clinical partners in the trial (see the “Treatment dosage” section). Patients recruited at the subacute phase (inpatients) will receive the rehabilitation treatment via RGS at the hospital, from recruitment until discharge. In addition, the patients in the intervention group are prescribed occupational or physical therapy following the local clinical practice of the rehabilitation unit from admission to discharge. Patients recruited after discharge (outpatients) will train with RGS at their homes daily for 3 months, including weekends. All the patients in the intervention group (inpatient and outpatient) receive the RGSwear at recruitment for up to 1 year post-recruitment.

The *control group* receives standard care provided to patients after clinical discharge, which varies according to the patients’ condition and the different standards of care practiced in each EU country [18]. Also, at discharge, patients are provided advice for continuing voluntary rehabilitation in their homes. Given the huge regional variability, a detailed description of standard care is not reported in this study. In the footnote, we have provided the links to the National Health guidelines for Spain^1^, France^2^, and Sweden^3^.

### RGS protocols and training

**Fig. 4:**
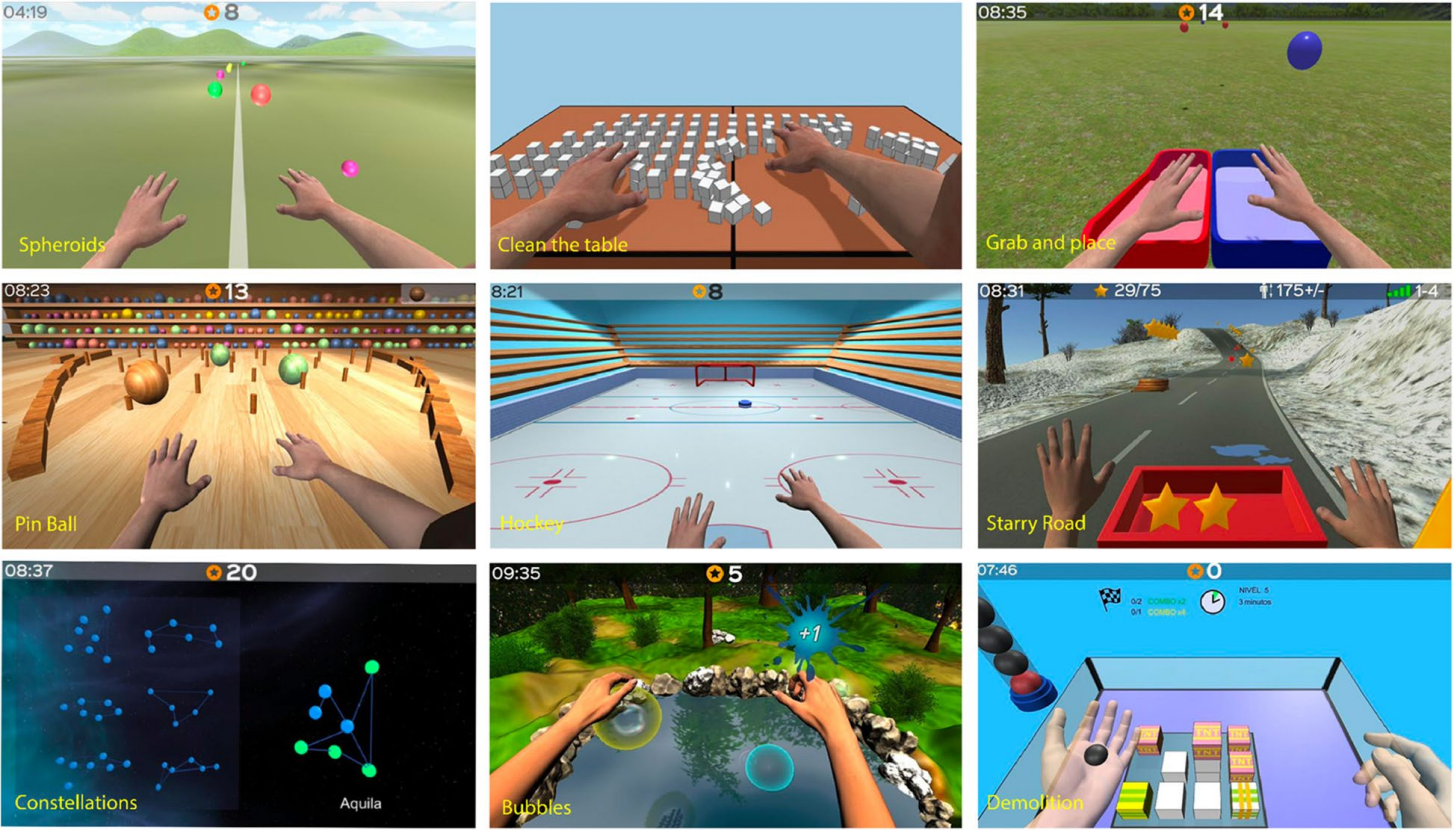
RGS training exercises*.* Examples of gamified exercises for the upper limbs with horizontal movement (spheroids, clean the table, pinball, hockey) or vertical movements (constellations, grab, and place) and exercises for the hands (bubbles, demolition)

The clinicians at each hospital have access to the RGS Medical Information Management System (MIMS), where they can select the training protocols and schedule the exercises for each patient. Training includes motor exercises for the upper limbs and hands such as reach, grasp, release, finger flexion, and pronation/supination. The exercises are combined with cognitive components (see Fig. 4). A variety of gamified exercises is proposed to ensure adherence to the treatment. The patients observe their movement performed by the virtual arms from a first-person perspective in a VR environment while performing the exercises in a horizontal (arms on the table) or vertical (arms up in the air) space. The patients’ movements (kinematics) and performance (gamified exercises) are recorded and stored in a secure remote database for future analysis (see the “Data collection and management” section).

### Treatment dosage

The RGS@home group follows a standardized intervention schedule as follows:

1. Daily training, including weekends.
2. Recommended training time is 24–28 min per day.
3. The number of exercises per day is between four and nine.
4. The duration of each exercise should be about 6 min.
5. At least one exercise should be changed every 2 weeks.
6. Patients are free to train more than prescribed.

After finishing the daily sessions prescribed by the therapists, patients are allowed to continue using the RGS protocols and repeat as many exercises as they want during the rest of the day. In addition, the RGSwear reminds the patient every other day to perform a task to move the paretic arm called circle drawing. We aim to correlate this movement with the Fugl Meyer Scale. This task consists of making several horizontal and vertical circles (clockwise and anticlockwise) on a table (without gravity) then the same task vertically in a non-obstructed space against gravity (see Fig. 1).

### Adherence to treatment

It is a common problem that patients sometimes lack the motivation to follow their training program when there is no direct supervision [19]. With RGS@home and RGSwear, the patient is continuously monitored, and the clinicians can detect low adherence and contact the patients. To further improve adherence, the exercises of the RGS-based training are designed to include gamification and motivational factors to avoid monotonous training. In addition, the AI component of RGS adapts the exercises to the patient’s abilities (increases or decreases the difficulty), thus avoiding frustration or boredom and facilitating patients’ adherence. Patients are free to leave the study at any time and will not be included in the study if they stop using RGS@home for more than five consecutive days. Patients are also free to participate in leisure activities. However, they should not participate in other clinical trials investigating rehabilitation approaches or technologies.

## Data collection and management

The clinical outcome measurements and the patients’ data are collected by each participating hospital by blinded clinicians and logged in the eCRF. Data obtained through the interaction with the RGS ecosystem is automatically logged in the MIMS. The data in the eCRF is securely stored and anonymized (patients are assigned an ID), and monitoring is organized by each clinical partner independently, adhering to local standards and procedures. To ensure all data is logged correctly and no duplicates exist, the RGS@home eCRF process automatically checks to ensure that the logged scores of the clinical scales and tests are valid. Two workshops have been organized with the study coordinators to understand how the assessments are performed and the data collected.

The data obtained from the interaction with the RGS ecosystem (kinematic data, prescriptions, protocol events, and use) is automatically stored in the MIMS, a secured cloud-based database that the participating hospitals can access via a web browser with a username and password. Within the MIMS, and separately from the eCRF, the participating hospitals can create profiles (anonymized) for each patient, determine and manage the prescriptions of exercises, and track the patient’s progress through dashboards, graphics, and analytic reports. The sponsor can remotely access the password-protected data in the MIMS to analyze the interaction with the RGS system.

### Data monitoring

The eCRF is provided by an external Contract Research Organization or CRO (SAIL, Barcelona) independently of the study sponsor. The CRO provides technical support and ensures that the data is safely stored on a secured server protected against unauthorized access. The CRO ensures that the eCRF was created according to the project’s needs by including mechanisms that ensure that the data is logged correctly (i.e., cannot exceed the scores’ max values), ranges are provided, or additional fields are provided to add information that ensures proper recording and understanding). Each clinical partner has access to the eCRF to enter the data collected and is responsible for correctly logging the data collected from their patients and ensuring the monitoring and auditing of that data. Harmonization workshops have been organized to ensure that data is assessed homogeneously across the clinical partners.

The CRO agrees that data can be extracted from the eCRF by the sponsor four times a year to perform preliminary analyses and comply with the trial’s reporting requirements. The anonymized data is shared only between the sponsor and the analytics partner responsible for data analysis.

## Statistical method

We will perform mainly between-subject analysis by comparing the mean outcomes of the experimental group with the mean outcomes of the control group. To analyze the clinical outcomes related to disability and impairment, we will calculate the normalized improvement, which shows improvement or decline in proportion to the maximum score possible in the respective scale, therefore avoiding ceiling effects. Depending on whether the data is following a normal distribution, we will use parametric or non-parametric statistics. In the case of non-normal data, medians will be reported. We will perform an intention-to-treat analysis. The last observation carried forward analysis will be performed alongside a complete-case analysis to account for missing data.

To address the *primary outcome on disability and impairment*, a between-subject analysis will be used to compare the change in disability (Barthel Index (BI)) between the experimental and control groups at baselines 3, 6, and 12 months.

For the *secondary outcomes* on usability and impairment and other planned comparisons, we will perform the following analysis (for abbreviations, see Table 2):

Changes in independence (CAHAI), participation (SIS), depression (HDRS) fatigue (FSS), pain (VAS), spasticity (mAS), and impairment (BI and FM-UE) are analyzed from baseline to months 3 and 12.

We will also look at the change in grip force and correlate it with FM-UE, CAHAI, and BI from baseline to 3 months and 12 months. The outcomes of this measure are important to evaluate the clinical effectiveness of the RGS intervention to reduce disability and impairment. To measure quality of life, we compare the group changes in the quality of life (SSQOL) and well-being (SF-36) from baseline to 3 months and 12 months. We will also compare well-being (SF-36) at 6 months.

For the additional variables shown in Table 3, we will proceed as follows:

*Readmission*: We compare between the groups the number of outpatients that return to the hospital after discharge to at-home status, including readmission due to complications unrelated to stroke, from baseline to 3, 6, and 12 months. We also compare the days of hospitalization and their associated costs. Here, we will take into account the presence of CVA risk markers as covarying variables. The readmission outcome is used to evaluate the effectiveness of the RGS intervention in reducing, or at least not increasing, a hospital’s readmission after discharge compared to standard care.

*Cost of treatment*: We compare the total cost of rehabilitation therapy/hour provided at the hospital or in the community and transportation costs/hour between the hospital and home at 3 months and after 12 months. We also compare days/hours of hospitalization and whether additional therapy, not replacing the RGS-based training (e.g., magnetotherapy, electrotherapy, thermotherapy), was provided. These outcomes allow us to evaluate the RGS’s effectiveness to reduce the cost of treatment.

*Quality of life*: We will compare between the groups the number of falls reported at 3 months and 12 months. In addition, we will compare how many patients were able to return to work and how many hours professional care was provided at 3 and 12 months. These outcomes facilitate the evaluation of the impact of the RGS ecosystem on quality of life and evaluate any risks arising from using the RGS system at home.

*Acceptance*: We will assess the RGS@home use (hours) after 3 months, 6 months, and 12 months. In both groups, we will keep track of the dropouts of the study. Specifically, in the intervention group, we will analyze the use of RGS at the hospital and home and the use of RGSwear. Concerning RGS@home, we will analyze the support needed (hours and visits to the patient’s home, replacement of materials, instructions, troubleshooting, etc.) and any incidents reported by the users. Also, we will analyze the patient’s evaluation of the System Usability Scale. The outcomes of this domain are important to evaluate the acceptance of the RGS ecosystem within the experimental group and the feasibility of the system for future use.

Lastly, we will analyze the kinematic data obtained from the motion tracking devices and the RGS training exercises. We will estimate the change in clinical scales from changes in kinematics and performance according to a data analysis regression pipeline that we have previously developed [17].

### Harms

The RGS system has obtained approval as a medical device that can be safely used in humans (approval obtained by *Agencia Española de Medicamentos y Productos Sanitarios, record number 792/20/E.C., and registered in EUDAMED, CIV-21-03-035989*). The approval process required an in-depth risk-benefit analysis, which classified the RGS system as a low-risk device. Nevertheless, we will collect and analyze all incidents, falls, reasons for readmissions, and reasons for dropout during the study, whether related or unrelated to the use of the devices. We do not foresee any reason to stop the trial. However, if any major incidents occur that pose a risk to the participating patients, the project leader can stop the trial.

### Auditing and protocol amendments

The clinicians of each participating hospital are responsible for adhering to the trial protocol and reporting any deviation or request for modification to the CRO and ethical committee.

Any deviations from the protocol will be fully documented using the eCRF.

### Ethics and confidentiality

#### Research ethics approval

The local ethical committee approved the study protocol, the assessment tools, and the informed consent form of each participating hospital:

Comité de Ética de la investigación con medicamentos CEIM for Parc Sanitari Sant Joan de Déu (ES) (https://www.sjdrecerca.org/es/investigacion/ceim/), Comité de Protection des Personnes SUD-EST II for Limoges (FR) (https://www.cppsudest2.fr/protocole_categorie_1.html), and Etikprövningsmyndigheten for Uppsala (SE) (https://etikprovningsmyndigheten.se/).

#### Consent or assent

The clinicians in charge of recruiting and screening the patients at each hospital are responsible for providing the informed consent form in paper to the possible candidate before any baseline evaluation is performed. The consent form contains all the information on the data collected and informs the patient that they can exercise their right of access, modification, opposition, and cancelation of data at any time. It also informs the patients that they can withdraw from the study at any point in time. On the consent form, participants will be asked if they agree to the use of their data should they choose to withdraw from the trial. Participants will also be asked for permission for the research team to share relevant data with people from the Universities taking part in the research or from regulatory authorities, where relevant. There is no anticipated harm (see also the “Harms” section) and compensation for trial participation. This trial does not involve collecting biological specimens for storage. The personal data will be logged into the eCRF system anonymized (patient ID).

#### Confidentiality

*Personal data* from the participating patients are considered strictly confidential according to current legislation on data protection (*Organic Law 3/2018 on 5 December of Personal Data Protection and Guarantee of Digital Rights, LOPD-GDD; the General Data Protection Regulation of the European Union, RGPD-UE, 679/2016 on 27 April 2016, and repealing Directive 95/46/E.C.; Regulation (E.U.) 2018/1807 on 14 November 2018 on a framework for the free flow of non-personal data in the European Union*).

*Non-personal data* obtained from the interaction with the RGS ecosystem is stored in a file after each training session (when starting and closing the application). Each patient is assigned generic login data (username and password) in each hospital via the MIMS. The non-personal data file is stored with the generic username and a session number. The patient’s identity cannot be inferred from the username or the session number, nor is the IP address or other personal data stored in the MIMS. All equipment is reset, and any local files are deleted before a patient receives the RGS set-up. The responsible for the storage of non-personal data is the technical partner in the study, who signed a confidentiality agreement. Backup and recovery processes are automated, performed weekly, and checked by the technical partner every 6 months.

The personal and non-personal data, whether on paper or in the eCRF, will be stored in their respective location 5 years after the completion of the study. All data obtained is limited to the patient’s participation in this trial and can only be used for research purposes.

#### Access to data

As it is a collaborative and multicentric European project, the interim and final trial datasets can be shared for scientific purposes between the RGS@Home collaborator. The sponsor will be responsible for collecting and transferring these data between collaborators. In this case, the data transferred will be anonymized or aggregated data. Each receiving partner ensures that the data will be stored safely on servers, with security measures, and within the European Union. All participating or collaborating centers sign the corresponding confidentiality agreement within the consortium agreement, following European regulations.

#### Dissemination policy

No preliminary clinical results will be published before the end of the trial, which is projected to be in the summer of 2023.

The datasets analyzed during the current study and statistical code are available from the corresponding author on reasonable request, as is the full protocol.

## Discussion

The RGS@home trial is designed to evaluate the effectiveness, acceptance, usability, and cost-effectiveness of a science-based ICT solution for rehabilitation (RGS) that combines brain theory, cloud computing, and VR. The RGS system targets motor and cognitive recovery after stroke. RGS is an integrated platform to deliver personalized treatment supporting the full treatment continuum, suitable for all end-users, i.e., patients, carers, and clinicians. The RGS@home trial validates an e-health approach towards neurorehabilitation that accompanies patients during their journey to recovery from the clinic to their homes, with an emphasis on counteracting disability and impairment and improving quality of life. The trial targets to demonstrate the superiority of the RGS intervention when combined with standard care compared to standard care alone while being usable and well accepted.

The trial includes so far one-third of the total number of patients. While the main results of the trial will be published in 2023, intermediate observations on the use of the system suggest that the patients in the intervention group use RGS@home exceeding the prescribed daily usage. They also enjoy the gamified VR-based training and can easily follow the training prescribed by the therapists. Thus, our preliminary observations indicate that the RGS@home solution is well received by the patients. The clinicians across the three European hospitals find it easy to use for treatment prescription, patient monitoring, and data management. We foresee that providing therapy via the RGS system at the patient’s home will reduce the risk of readmission and prevent deterioration. We hypothesize that the additional therapy time at home positively influences arm use and reduces impairment (confirming a previous pilot study by [15]).

The proposed solution promises to be highly scalable; hence, many patients can be benefiting simultaneously without overhead on the health care personnel. In addition, it will be cost-effective since it comprises off-the-shelf devices that are ubiquitous in our society. The reduction of readmissions and prevention of deterioration should reduce transportation costs, therapy, and hospital stays. Most importantly, this AI-based training solution allows extending the hospital’s rehabilitation service treatment to the patient’s home, which will result in cost reduction for hospitals and families.

In addition, the RGS@home platform allows for remote monitoring and assessment so that clinicians and therapists can follow the patients’ progress and adjust the training as needed. We expect our results to show increased adherence to treatment and further optimizations in care and quality of life.

Lastly, the multi-modal data obtained in the RGS@home trial will advance our understanding of long-term recovery patterns in chronic stroke patients, which will further facilitate reforming stroke rehabilitation towards a leaner, more cost-effective, and evidence-based approach that benefits both patients, health care providers, and society.

## Data Availability

The data will not be publicly available given that the trial is still not concluded. However, it may be made available to interested third parties upon request to the consortium and only for scientific purposes. Each partner should inform the consortium about any other planned publication, presentations, or other dissemination activities regarding the project. No preliminary clinical results will be published before the end of the trial.

## Abbreviations

RCT: Randomized clinical trial
ICT: Information and communication technology
RGS: Rehabilitation gaming system
AI: Artificial intelligence
ADL: Activity of daily living
VR: Virtual reality
MIMS: Medical Information Management System
eCRF: Electronic case report form

## Footnotes

1 Ministry of Health S, Services and Equality. Stroke Strategy of the Spanish National Health System. 2012. https://www.sanidad.gob.es/fr/organizacion/sns/planCalidadSNS/docs/Stroke_Strategy.pdf

2 Rééducation à la phase chronique d’un AVC de l’adulte: Pertinence, indications et modalités - Note de cadrage. https://www.has-sante.fr/upload/docs/application/pdf/2020-02/18rbp321_rpc_cadrage_reeduc_avc_29-10-19.pdf

3 Performance assessment of stroke care. Adherence to National Guideline. 2018. https://www.socialstyrelsen.se/globalassets/sharepoint-dokument/artikelkatalog/nationella-riktlinjer/2019-1-18-eng.pdf
